# Event and Comment

**Published:** 1913-03-15

**Authors:** 


### DENTAL REGISTER.

**Vol. LXVII. March 15, 1913. No. 3**

### EVENT AND COMMENT.

INSTITUTE OF DENTAL PEDAGOGICS. The twentieth annual meeting of the Institute of Pedagogics was held at Pittsburgh January 28th to 30th and was largely attended by representatives from nearly every dental college in this country and Canada. It is surprising that this organization keeps up its interest in the way it does. Ten years ago we thought it would not continue to attract the attention of our teachers much longer; but if there is any tendency to loose interest it was certainly not apparent at this meeting. The programme was full of interest and there was not time for all the discussions. The character of the papers read and the discussions were of a high order, although in some of the papers the pedagogic element was overshadowed by the artistic treatment. This was, however, corrected to a large extent in the discussions which always assumed the practical form. A purely pedagogic treatment of all subjects would probably be tiresome and after all this latitude can be indulged for the sake of keeping up the interest. Every paper on the programme was presented and nearly every man on the programme appointed to formally discuss the papers was present.

The social features were exceedingly interesting and while not obtrusive or absorbing they kept the pedagogues busy every minute. The Dental School of the University of Pittsburgh has just moved into its new building which is located on the highest point of the campus, which is so high as to take it out of the realm of smoke, for which the city is famous, thus assuring good light and wholesome exercise for the students. The building is admirably adapted to its purpose, and is well equipped with modern appliances for teaching dentistry. The location of this school and its excellent facilities with a faculty of young and progressive men ought to make it grow to become one of our largest and best teaching institutions. We predict there will be a good sized addition to this building in the next ten years. The building is located in the finest section of Pittsburgh, close to the great Carnegie Library and Museum and the Carnegie Technical Institution. The atmosphere is one of culture and technical attainments and there should be an educational development here worthy of our best American Universities. The meeting at this place made a deep impression on the minds of the delegates and we are confident they all returned to their schools with new inspirations and renewed energies for better service. We do not know of a dental organization that is doing better work than this one and we hope it may have a long life and a place of influence commensurate with its importance. An effort to change the name to the National Association of Dental Teachers was lost and we believe it is for the best. The name Institute of Dental Pedagogics is dignified and significant and there is no good reason for changing it for one that has no better claim than that it is more euphonius.

ANNUAL CLINIC OF THE CHICAGO DENTAL SOCIETY. On the last day of January and the first day of February the Chicago Dental Society gave its great clinic and a greater banquet to one of its most distinguished members Dr. Trueman W. Brophy.

The first day was given over to an exhibition of manufacturers of dental supplies in Northwestern Dental School and an address in the evening by Dr. Charles H. Mayo, the distinguished surgeon of Rochester, Minn., on the subject of "The Relation of Local Foci of Infection to General

Systemic Conditions." The address was very interesting and profitable and was ably discussed by Drs. Herrick, Gilmer and Lewis.

The second day was devoted to clinics on almost every conceivable subject, nearly a hundred of them. In fact there were so many of them and such a crowd that they could not be seen to advantage by any one. There are disadvantages sometimes in bigness and this seemed to us the chief drawback on this occasion.

The Brophy banquet in the evening was the most elaborate affair of its kind we have ever seen and we doubt if anything equalling it has ever been done, even in Chicago. The large banquet hall in the Hotel La Salle was completely filled with dentists from all over this country and there were several from far distant countries. There were speeches, telegrams, cablegrams, presents and testimonials from nearly everybody of note in the dental world. Then there were speeches and the speeches were good and most laudatory, from men both in and out of the profession.

We can not understand how anyone could undergo such an outpouring of appreciation for faithful and devoted service to any cause without being absolutely overcome. But Dr. Brophy was big enough in mind and heart to accept it all comfortably and we have no doubt he will feel happier that he knows what his conferree's think of his faithful service to their cause. It does not happen to every man to have such a tribute while he is still in active service and it certainly should make us all feel that life is really worth living, when it even occasionally meets such a cordial and spontaneous approval. The members of the Committee having in charge this meeting must have done extra time in completing arrangements, as every detail was executed with precision and a completeness that was marvelous. We wonder more than ever when some Chicago dentists get time to practice, or if they ever sleep, we know they eat.

